# Patient safety in primary care: a survey of general practitioners in the Netherlands

**DOI:** 10.1186/1472-6963-10-21

**Published:** 2010-01-21

**Authors:** Sander Gaal, Wim Verstappen, Michel Wensing

**Affiliations:** 1 Scientific Institute for Quality of Healthcare, Radboud University Nijmegen Medical Centre, P.O. Box 9101, 114 IQ healthcare, 6500 HB Nijmegen, The Netherlands

## Abstract

**Background:**

Primary care encompasses many different clinical domains and patient groups, which means that patient safety in primary care may be equally broad. Previous research on safety in primary care has focused on medication safety and incident reporting. In this study, the views of general practitioners (GPs) on patient safety were examined.

**Methods:**

A web-based survey of a sample of GPs was undertaken. The items were derived from aspects of patient safety issues identified in a prior interview study. The questionnaire used 10 clinical cases and 15 potential risk factors to explore GPs' views on patient safety.

**Results:**

A total of 68 GPs responded (51.5% response rate). None of the clinical cases was uniformly judged as particularly safe or unsafe by the GPs. Cases judged to be unsafe by a majority of the GPs concerned either the maintenance of medical records or prescription and monitoring of medication. Cases which only a few GPs judged as unsafe concerned hygiene, the diagnostic process, prevention and communication. The risk factors most frequently judged to constitute a threat to patient safety were a poor doctor-patient relationship, insufficient continuing education on the part of the GP and a patient age over 75 years. Language barriers and polypharmacy also scored high. Deviation from evidence-based guidelines and patient privacy in the reception/waiting room were not perceived as risk factors by most of the GPs.

**Conclusion:**

The views of GPs on safety and risk in primary care did not completely match those presented in published papers and policy documents. The GPs in the present study judged a broader range of factors than in previously published research on patient safety in primary care, including a poor doctor-patient relationship, to pose a potential threat to patient safety. Other risk factors such as infection prevention, deviation from guidelines and incident reporting were judged to be less relevant than by policy makers.

## Background

Patient safety has received increased attention worldwide [1]. The focus of research is mostly upon hospital care [2], although most patients attain their healthcare in primary care settings, particularly in countries with a strong primary care system [3]. Primary care has been found to be relatively safe although incidents do occur in this setting as well [4]. The occurrence of incidents in primary healthcare has been estimated to be somewhere between 5 and 80 times per 100,000 consultations [5]. Different definitions of patient safety and a patient safety incident have been published. A working group from the World Health Organization, for example, has defined a patient safety incident as an event or circumstance which could have resulted, or did result, in unnecessary harm to a patient [6]. Such a definition is useful but it does not specify which components of healthcare delivery may be related to patient safety.

In primary care practice, consideration of patient safety is mostly associated with the reporting of incidents and specific aspects of the delivery of healthcare such as medication safety and the prevention of infection [7]. However, in a recent interview study with physicians and nurses in primary care, the scope of patient safety was found to be much broader than the aforementioned [8]. The views of health professionals should thus be sought to identify what risk and safety means in actual practice. In the present study, general practitioners (GPs) were surveyed to gain better insight into what they consider unsafe practices and what they judge to be risk factors for patient safety in primary care.

## Methods

### Study design and setting

A web-based survey was conducted in a sample of GPs in the Netherlands. All of the GPs from the Nijmegen University Network of General Practitioners (NUHP) were invited to participate in the study (n = 132). The NUHP is a network of Dutch general practices affiliated with the Radboud University Nijmegen for research purposes, the education of medical students and the training of vocational trainees. The practices thus came from a wide region around the city of Nijmegen, included both rural and urban practices and did not differ from the Dutch average with respect to various demographic characteristics (Table 1). After an invitation to participate in the study was sent to all the GPs, they were e-mailed the survey using an internet survey software program; those GPs with no email address were sent a paper version of the survey. Non-respondents were sent a second invitation one week later and a third invitation one month later. The ethical committee of the Radboud University Nijmegen Medical Centre approved the study.

**Table 1:** Demographic characteristics of the GPs who completed the survey

	*Our study*	***Netherlands average ***[24]
Sex no. (%)		
Male	44 (64.7%)	65%
Female	24 (35.3%)	35%

Mean Age (years) ± SD	48.4 years (± 7.5)	46.6 years

Practice		
Solo	5 (7.4%)	
Duo	20 (29.4%)	
Group	41 (60.3%)	
Unknown	2 (2.9%)	

Mean years of experience ± SD	17 (± 9.6)	

Mean FTE ± SD	0,73 (± 0.20)	

Practice area		
<5000 habitants	10	
5.000 - 30.000 habitants	29	
30.000 - 100.000 habitants	13	
>100.000 habitants	16	

### Design of the survey

The content of the survey was derived from the results of an interview study undertaken with physicians and nurses to explore what constitutes 'patient safety' in primary care [8]. The semi-structured interviews yielded a wide range of items considered relevant for patient safety. The points included -for instance- safe electrical sockets, or a definition like answer as 'do not harm the patient.' A set of salient points was selected next and put into a survey which was then reviewed by three experts (i.e., experienced GPs also involved in patient safety research). The web-based survey included brief descriptions of 10 clinical cases (Table 2) and a list of 15 factors (Table 3) to be evaluated by the respondents in terms of placing patient safety at risk. A definition of patient safety was not provided with the survey.

**Table 2:** Clinical cases

	*Theme*	*Patient safety judged to be at risk (% GPs)*
1. A mother calls about her three-year-old daughter who has a fever. The medical assistant who handled the call did not detect any alarming symptoms and provided advice in keeping with guidelines. Given that it was very busy in the practice, the medical assistant did not enter the advice provided into the patient's electronic medical record.	Content of medical record	85.5%

2. The electronic medical record of a GP produces a lot of medication interaction warnings. The GP often ignores these without reading the warnings carefully.	Medication	85.3%

3. A cardiologist prescribes a patient a new ACE inhibitor within the context of a clinical trial. The patient already has chronic kidney failure. The GP considers checking the suitability of the medication to be entirely the responsibility of the cardiologist and therefore takes no action.	Medication	76.5%

4. A GP prescribes a NSAID for a ankle distortion to a 70-year-old male with no GI complaints or other medicines for a period of three days. The GP does not give gastric protection.	Medication	73.5%

5. A practice does not discuss errors made in the practice on a regular basis. Errors are resolved on an ad hoc basis by the healthcare workers involved.	Error discussion	51.5%

6. A study shows a patient to have to wait more than 10 minutes to speak to a medical assistant on the regular practice telephone number 40% of the time.	Telephone accessibility	26.5%

7. There has been a miscommunication between medical assistant and patient with regard to appointment time; the patient does not show up for appointment. The GP does not know what complaint the patient was coming for or when the patient may show up.	Miscommunication	22.1%

8. A 65-year-old man wants to know his PSA level. He has no prior complaint and the family history is negative. The GP discusses the advantages and disadvantages of drawing the PSA. Despite the possible disadvantages, the GP decides to draw the PSA because the patient wants to know his PSA value	Preventive medicine	20.5%

9. A patient is admitted to the hospital with a perforated appendix. Earlier that day, the patient was seen by a GP. The GP gave clear instructions on when the patient should return to see him, and the patient indeed returned to see him.	Diagnostic process	17.6%

10. In a general practice, small surgical procedures which require suturing are done without sterile gloves.	Hygiene	10.3%

**Table 3:** Risk factors

	*Theme*	*Patient safety judged to be much/very much at risk (%GPs)*
1. Not keeping one's medical knowledge up-to-date	Knowledge	42.6%

2. Poor doctor-patient relationship	Communication	41.2%

3. Patient age >75 year	Age	41.2%

4. Language barrier between GP and a non-western immigrant	Language barrier	36.8%

5. Patient with more than 5 medicaments	Polypharmacy	33.8%

6. Patient who 'shops' between different GPs in the same practice	Different GPs	23.5%

7. No telephone triage	Triage	22.1%

8. Delayed receipt of information about patients from hospital	Lack of information	17.6%

9. Patient who frequently comes for medically unexplained complaints	Unexplained complaints	13.2%

10. Patient age >70 year	Age	10.3%

11. Patient with a chronic disease	Chronic disease	10.3%

12. Patient who has consulted more than twice during GP's office hours for the same complaint	Repeat visits	7.4%

13. Need to make an emergency visit during regular office hours.	Time pressure	7.4%

14. Deviation from guidelines provided by Dutch College of General Practitioners	Evidence based medicine	2.9%

15. Lack of privacy at reception or in waiting room	Privacy	0%

For each of the clinical cases, the respondent was asked to judge the impact of the specific situation on patient safety along a five-point Likert scale which ranged from 'patient safety not at all at stake' to 'patient safety is greatly at stake.' The respondent could also provide comments. For the list of potential risk factors, the respondent was also asked to judge these risk factors along a five-point Likert scale which ranged from 'no increased risk for patient safety' to 'greatly increased risk for patient safety.' Finally, the survey also included some questions to determine the demographic characteristics of the general practice.

The data were entered into SPSS 16.0 for analysis. The response frequencies for the GPs were calculated. No significant differences (p < 0.05) were found between the answers provided by the GPs from different geographic areas, male versus female GPs or different aged GPs (i.e., those 50 or over versus those under 50). The comments provided by the GPs were analyzed qualitatively per clinical case. One comment per clinical case was used as enlightening quote.

## Results

The survey was completed by 68 of the 132 GPs we approached, which is a response rate of 51.5%. Of the 68 respondents, 65% was male; 35% was female. The mean age was 48.4 years. This is comparable to the national population of Dutch GPs (Table 1). A total of 146 comments were provided by the GPs on the 10 clinical cases presented to them. The content of the comments are noted with the clinical cases.

### Judged safety of specific clinical practices

Five of the 10 clinical cases presented to the GPs were judged to be unsafe by a majority of them (>50%). The other five cases were judged to be safe by a majority of the GPs (Table 2).

### Medical record systems

#### Always note the advice given. A second call must be treated differently. (GP 27)

The case in which the practice assistant does not record the advice provided to the mother of a child with a fever and the case in which the GP overrules the medical warnings produced by the computer were judged to be a threat to patient safety by the highest number of GPs; 85.5% and 85.3% of the GPs judged patient safety to be at risk in these cases, respectively. Many of the GPs used the comment box to also note that they, themselves, recognized the situation from actual daily practice in both cases as well.

### Medication

#### The GP must consult the cardiologist with regard to this possible interaction when he judges the risk for the patient to be high. (GP 38)

The case of the GP not acting with regard to a possible interaction with medication prescribed by a specialist was judged to constitute a major threat to patient safety by 76.5% of the GPs and thus as the third most critical clinical case. Many of the GPs explicitly stated that it is also a responsibility for the practicing GP to take action even when he or she did not prescribe the medication.

The case in which a NSAID is prescribed for a few days to an elderly patient but without gastric protection was similarly judged to be unsafe by 73.5% of the GPs and thus as a critical clinical case. The GPs repeatedly noted that it is better to provide precautionary gastric protection in all elderly patients, regardless of the presence of gastric complaints or not.

### Error discussion

#### A missed chance to learn from errors made. (GP 30)

The case in which errors made in the practice are not discussed on a regular basis was judged by 51.5% of the GPs to place patient safety at risk. Most of the comments concerned the fact that regular discussion of errors with the whole practice team allows the practice to learn from mistakes.

### Telephone accessibility

#### There is almost always an emergency line to bypass the waiting time; this is a must. (GP 45)

The case in which some 40% of patients had to wait more than 10 minutes for contact via the telephone on a regular basis [9] was judged as unsafe by only 26.5% of the GPs. However, almost every GP commented in this connection that there had to be an emergency line.

### Miscommunication

#### You can't call back every patient; especially young patients often forget their appointments. (GP 46)

Only 22.1% of the GPs judged a patient not showing up for an appointment when the purpose of the appointment is further unknown to be unsafe. Many of the GPs commented that showing up for an appointment is also the responsibility of the patient. Some of the GPs mentioned that their own practices used telephone triage, which means that the practice always knows what the patient made an appointment for.

### PSA testing

#### Only if the value of the test is thoroughly explained. (GP 38)

PSA screening for prostate cancer in a 65-year-old patient with no current complaints was judged as placing patient safety at risk by 20.5% of the GPs. The question, as stated by one GP, is whether the patient's fear of cancer outweighs the disadvantages of a biopsy prompted by a false-positive PSA outcome. Other GPs stated that the patient has a right to preventive screening; it is the task of the GP to explain the pros and cons of such screening.

### Diagnostic process

#### This is normal and good practice; you can't send everyone to the hospital. (GP 17)

In the case of the patient seen by the GP for abdominal pain and later admitted to the hospital with a perforated appendix, 17.6% of the GPs judged the described diagnostic process to constitute a threat to patient safety. The majority of the GPs judged the described course of events to be 'all in the game' - one cannot predict the future. In the opinion of many of the GPs, patient safety is not at risk when adequate physical examination is undertaken and appropriate conclusions are drawn.

### Hygiene

#### There are healthy bacteria in primary care; in hospital, there are more pathogens. (GP 27)

The case of suturing in the primary care practice without the use of sterile gloves was only judged to constitute a threat to patient safety by 10.3% of the GPs. This is least of all the clinical cases. Many of the GPs commented that they almost never saw infections in their practices when they used non sterile gloves. Some of the GPs reported not using sterile gloves while suturing for more than 25 years and not seeing any secondary infection.

### Potential risk factors

The percentages of the GPs who scored the potential risk factors as constituting 'much' or 'very much' of a risk to patient safety were next calculated (Table 3). The highest ranked factors were not keeping up one's medical knowledge (42.6%), a poor doctor-patient relationship (41.2%) and patient age over 75 years (41.2%). The existence of a language barrier (36.8%) and polypharmacy (33.8%) were also judged to place patient safety at risk although somewhat less than the aforementioned factors. Patients presenting with unexplained symptoms and repeat visits by patients for the same symptoms were not viewed as much of a risk factor by the GPs (13.2% and 7.4%, respectively). Deviation from the evidence-based guidelines provided by the Dutch College of General practitioners (which is a well-known primary care organization in the Netherlands which has made evidence based guidelines on the most prevailing complaints in primary care) was judge to be unsafe by only 2.9% of the GPs, and none of the GPs correlated lack of privacy in the waiting room with patient safety.

## Discussion

The present survey is -to our knowledge- one of the first to examine physicians' views on patient safety during daily primary care. The clinical cases judged to be unsafe by a majority of the GPs concerned the use of the medical record system and the prescription and monitoring of medication. The clinical cases judged to pose little or no threat to the safety of primary care patients concerned hygiene, diagnostic procedures, prevention and communication. The aforementioned clinical cases also correlate with a taxonomy of patient safety in primary care [10].

The potential risk factors judged to be most unsafe for primary practice were a poor doctor-patient relationship, insufficient maintenance of the GP's medical knowledge and a patient over 75 years of age. Language barriers and polypharmacy were also frequently judged to constitute risk factors for patient safety in primary care. Remarkably, deviation from evidence-based guidelines and privacy in the waiting room were not perceived as threats to patient safety by the GPs in our study.

None of the clinical cases were uniformly assessed as safe or unsafe by the GPs; considerable variation in the views of the GPs was observed. In a different study in which GPs were presented five cases of possible clinical error, 47% to 100% of the GPs judged an error to have been made [11]. The five cases included a broken tube during lab testing and the incorrect interpretation of lab results by the GP (i.e., cases in which the primary care clearly went wrong). The option to comment further on the clinical cases was often used in our survey, which suggests that judgments of patient safety -just as definitions of medical error- greatly depend upon individual attitudes and may thus be arbitrary to a considerable extent.

### Perceptions of patient safety

Out of the 10 clinical cases responded to by the GPs in our study, failure to record or inadequate notation of information in the medical records of patients was judged to constitute the greatest threat to patient safety. This finding is consistent with the results of other studies which show missing information to be common and possibly harmful for patients in primary care [12]. One of the lessons from the *Threats to Australian Patient Safety *(TAPS) study, moreover, is the importance of complete and accurate medical records. Errors can arise from missing clinical information (missing lab results) and/or suboptimal recording of contacts within an episode of care [13]. Our findings confirm this. The GPs in our study considered good record keeping to be highly important for patient safety.

Medication safety was also perceived by the GPs in our study to be highly critical for the safety of their primary care patients. This included the clinical cases of overruling medical alerts, nonresponse to possibly dangerous interactions of hospital prescribed medications and the prescription of a NSAID without gastric protection for an elderly patient. Medication safety is probably the best studied aspect of patient safety. The results of a recent study in the Netherlands, for example, showed adverse drug events to be an important cause of unplanned hospitalization with almost 50% of the hospitalizations potentially preventable [14]. The clinical case which concerned the overruling of medical warnings generated by an electronic dossier in our study was judged by 85% of the GPs to be quite risky; nevertheless, recent research shows clinicians to override most medication alerts, which suggests that the system does not function adequately and protect patients [15]. In a different study, few physicians were found to change their prescriptions in response to drug allergy or interaction alerts [16]. The GPs in our survey study placed a greater emphasis on medication monitoring than in our interview study [8].

The case in which a practice did not discuss errors on a regular base was only judged to pose a moderate risk for patient safety. The reporting of incidents can help healthcare professionals learn from mistakes and thereby improve the delivery of healthcare in the future [17]. Broad implementation of incident reporting is one of the targets of health policy in many countries including the Netherlands. However, in the present study, only 50% of the GPs viewed this as an issue for patient safety, which appears to be in line with research providing limited evidence for the effectiveness of incident reporting to improve patient safety. Of course, our finding may also indicate reluctance on the part of GPs to undertake incident reporting due to time constraints and/or the challenge which such reporting could present for their professional competence.

Telephone waiting time was judged low in terms of posing a threat to patient safety. While the Dutch Healthcare Inspectorate [9] reports a wait of 2 minutes for contact via a regular telephone line to be acceptable, the GPs in our study generally considered a wait of as much as 10 minutes to not constitute a threat to patient safety. The GPs in our survey study may have judged a long wait as less than optimal but not unsafe although this contradicts the results of our interview study in which both doctors and nurses suggested that telephone accessibility of the primary care practice is important for patient safety [8]. Accessibility may, of course, refer to the availability of an emergency telephone line, which almost all GPs consider a necessity, but the Dutch Healthcare Inspectorate reports more than 25% of patients calling an emergency telephone line to not receive an answer from the primary care practice [9].

The clinical case judged to pose the least of a threat to patient safety in the present study was suturing without sterile gloves. Many of the GPs explicitly stated that no use of sterile gloves is safe - despite a Dutch clinical guideline which says that the use of sterile gloves is mandatory for the prevention of infection [18]. Hand hygiene is an area in which physicians have been found to be remarkably resistant to procedures recommended for the prevention of major infection [19], and our own findings are thus consistent with this. A wide range of barriers to change in the direction of prevention has also been identified and found to include, among other things, insufficient knowledge of evidence regarding infection prevention and insufficient availability of the necessary devices.

### Perceptions of risk factors

Failure to keep one's medical knowledge up-to-date scored high as a risk factor for patient safety. Medical knowledge is of obvious importance, and insufficient knowledge can result in inadequate decision-making for both diagnostic and treatment purposes [20]. Interestingly, a poor doctor-patient relationship scored equally high as a risk factor for patient safety. A poor doctor-patient relationship can have negative outcomes for patient satisfaction, treatment compliance and even the health status of the patient [21]. The diagnostic process can also be complicated by a poor doctor-patient relationship and communication problems, with inadequate diagnosis as a result.

In contrast, deviation from evidence-based guidelines and hygiene (i.e., the case of suturing without sterile gloves) were not viewed as a major threat to patient safety by the GPs in our study. We can only speculate that physicians consider deviation from evidence-based guidelines as suboptimal treatment but not harmful to the patient. This suggests that undertreatment or failure to provide the treatment recommended by a guideline may not be part of the physician's concept of patient safety. It is also possible that physicians clearly see their deviation from evidence-based guidelines to be based upon adequate clinical decision-making and careful consideration.

### Strengths and weaknesses of this study

The response rate for this study was acceptable, but selection bias cannot be ruled out. In light of the involvement of all our respondents in the Nijmegen University Network of General Practitioners (i.e., training of medical students), the respondents in our study were perhaps more interested in patient safety than the average GP in the Netherlands. However, the demographic characteristics of the respondents in our study were representative for the population of GPs in the Netherlands and the answers provided by the GPs in our study did not differ systematically across subgroups. While the survey used in this study was not empirically validated, it was nevertheless based upon the results of interviews and the insights of experienced GPs with regard to the choice of clinical cases and potential risk factors. The primary care cases we presented as part of the survey were actually presented to us by the GPs in our previous interview study. Such cases indeed occur frequently in daily practice, which is supported by not only our own clinical experience but also the comments of the respondents in our survey study. That is, many of GPs used the comment box to explicate the score they assigned and a number of these comments indicated that the case in question was indeed a problem in their own clinical practice as well.

### Implications for future research

The results of this study highlight which aspects of general practice care are viewed as most important for patient safety from the perspective of the GPs themselves. Nevertheless, the scope of patient safety is broader than the perspective of only the GP [4]. The GPs in our study judged well-known medication factors (e.g., prescription and monitoring, adherence to alerts) as critical for patient safety but also less well-known factors such as a good doctor-patient relationship. The Manchester Patient Safety Framework for Primary Care is available to chart the safety of the healthcare culture [22]. However, for adequate implementation of such a monitoring system into primary care, it is important that what the GPs themselves consider most important for patient safety in actual practice be taken into consideration as well. Obviously, strategies to improve patient safety are needed. Organizational culture may play an important role in patient safety improvements [23]. It would be inappropriate to narrow down patient safety programs to the monitoring of medication and prevention of infection in primary care, for instance, but the necessary breadth poses a major challenge for the development of patient safety programmes and the actual measurement of patient safety because valid measurement and improvement trajectories require specificity. Further research should be conducted on the implementation of the present findings into useful patient safety programs. Finally, it might be useful to investigate the correspondence between the definitions and perception of patient safety provided by patients and GPs.

## Conclusions

The GPs in this study judged not keeping detailed and up-to-date medical records, not heeding electronic warnings and doctors responsibility as critical issues for patient safety. A poor doctor-patient relationship, failure to maintain one's medical knowledge and polypharmacy were scored highest as risk factors for patient safety. Guideline adherence, patient privacy and telephone waiting time scored low. The present findings have implications for the further study of patient safety and the improvement of primary care.

## Competing interests

The authors declare that they have no competing interests.

## Authors' contributions

SG developed the study, collected the data, performed the analyses, presented the results and conclusions and drafted the manuscript. WV contributed to the conception and design of the study and revised the manuscript. MW supervised the study, contributed to the conception and design of the study and revised the manuscript. All authors read and approved the final manuscript.

## Pre-publication history

The pre-publication history for this paper can be accessed here:

http://www.biomedcentral.com/1472-6963/10/21/prepub

## References

[B1] Donaldson SL An international language for patient safety: Global progress in patient safety requires classification of key concepts Int J Qual Health Care 2009 21 1 10.1093/intqhc/mzn056 19147594 PMC2638756

[B2] Stelfox HT Palmisani S Scurlock C Orav EJ Bates DW The "To Err is Human" report and the patient safety literature Qual Saf Health Care 2006 15 174 178 10.1136/qshc.2006.017947 16751466 PMC2464859

[B3] Starfield B Shi L Macinko J Contribution of primary care to health systems and health Milbank Q 2005 8 457 502 10.1111/j.1468-0009.2005.00409.x PMC2690145 16202000

[B4] Wetzels R Wolters R van Weel C Wensing M Mix of methods is needed to identify adverse events in general practice: a prospective observational study BMC Fam Pract 2008 9 35 10.1186/1471-2296-9-35 18554418 PMC2440745

[B5] Sandars J Esmail A The frequency and nature of medical error in primary care: understanding the diversity across studies Fam Pract 2003 20 231 236 10.1093/fampra/cmg301 12738689

[B6] World alliance for patient safety drafting group Towards an international classification for patient safety: the conceptual framework Int J Qual Health Care 2009 21 2 8 10.1093/intqhc/mzn054 19147595 PMC2638753

[B7] De Leeuw JBR Veenhof C Wagner C Wiegens TA IJzermans JC Schellevis FG de Bakker DH Patiëntveiligheid in de eerstelijnszorg: stand van zaken. [Patient safety in primary care: The current state of affairs] 2008 Utrecht, NIVEL

[B8] Gaal S van Laarhoven E Wolters R Wetzels R Verstappen W Wensing M Patient safety in primary care has many aspects: an interview study in primary care physicians and nurses J Eval Clin Pract in press 10.1111/j.1365-2753.2010.01448.x 20438606

[B9] Inspectie voor de Gezondheidszorg Telefonische bereikbaarheid van huisartsen moet sterk verbeteren. [Telephone accessibility of GPs has to improve significantly]. Den Haag 2008

[B10] Dovey SM Meyers DS Philips RL Jr Green LA Fryer GE Galliher JM Kappus J Grob P A preliminary taxonomy of medical errors in family practice Qual Saf Health Care 2002 11 233 238 10.1136/qhc.11.3.233 12486987 PMC1743626

[B11] Elder NC Pallerla H Regan S What do family physicians consider an error? A comparison of definitions and physician perception BMC Fam Pract 2006 7 73 10.1186/1471-2296-7-73 17156447 PMC1702358

[B12] Smith PC Araya-Guerra R Bublitz C Parnes B Dickinson LM Van Vorst R Westfall JM Pace WD Missing clinical information during primary care visits JAMA 2005 293 565 571 10.1001/jama.293.5.565 15687311

[B13] Makeham MA Bridges-Webb C Kidd MR Lessons from the TAPS study - errors relating to medical records Aust Fam Physician 2008 37 243 244 18398521

[B14] Leendertse AJ Egberts AC Stoker LJ Bemt PM van den Frequency of and risk factors for preventable medication-related hospital admissions in the Netherlands Arch Intern Med 2008 168 1890 1896 10.1001/archinternmed.2008.3 18809816

[B15] Isaac T Weissman JS Davis RB Massagli M Cyrulik A Sands DZ Weingart SN Overrides of medication alerts in ambulatory care Arch Intern Med 2009 169 305 311 10.1001/archinternmed.2008.551 19204222

[B16] Weingart SN Toth M Sands DZ Aronson MD Davis RB Phillips RS Physicians' decisions to override computerized drug alerts in primary care Arch Intern Med 2003 163 2625 2631 10.1001/archinte.163.21.2625 14638563

[B17] Fernald DH Pace WD Harris DM West DR Main DS Westfall JM Event reporting to a primary care patient safety reporting system: a report from the ASIPS collaborative Ann Fam Med 2004 2 327 332 10.1370/afm.221 15335131 PMC1466702

[B18] Dutch Working party on infection prevention(WIP) guideline for primary care http://www.wip.nl/free_content/Richtlijnen/1Huisartsen.pdf (accessed 6 May 2009)

[B19] Trampuz A Widmer AF Hand hygiene: a frequently missed lifesaving opportunity during patient care Mayo Clin Proc 2004 79 109 116 10.4065/79.1.109 14708954 PMC7094481

[B20] Makeham MA Mira M Kidd MR Lessons from the TAPS study - knowledge and skills errors Aust Fam Physician 2008 37 145 146 18345364

[B21] Ong LM de Haes JC Hoos AM Lammes FB Doctor-patient communication: a review of the literature Soc Sci Med 1995 40 903 918 10.1016/0277-9536(94)00155-M 7792630

[B22] National Primary Care Research and Development Centre, University of Manchester Manchester Patient Safety Framework for Primary Care http://www.nrls.npsa.nhs.uk/EasySiteWeb/getresource.axd?AssetID=60007&type=full&servicetype=Attachment (accessed 9 November 2009)

[B23] Scott T Mannion R Marshall M Davies H Does organisational culture influence health care performance? A review of the evidence J Health Serv Res Policy 2003 8 105 117 10.1258/135581903321466085 12820673

[B24] Hingstman L Kenens RJ Cijfers uit de registratie van huisartsen - peiling 2007. [Figures from the GP registration - survey 2007] 2007 NIVEL Utrecht

